# *Shewanella algae* Necrotizing Fasciitis and Septic Shock after Elective Cosmetic Surgery: A Potential Emerging Infection

**DOI:** 10.4269/ajtmh.25-0747

**Published:** 2026-08-13

**Authors:** Eryn Percenti, Rebecca Nowacki, Adam Fennell, Elora Friar, Anthony Cannella

**Affiliations:** ^1^ Department of Internal Medicine, Division of Infectious Disease and International Medicine, University of South Florida Morsani College of Medicine, Tampa, Florida, USA;; ^2^ Department of Internal Medicine, University of South Florida Morsani College of Medicine, Tampa, Florida, USA

## Abstract

*Shewanella* is a Gram-negative bacillus found in marine environments throughout the world. Its role in human pathogenesis has mostly been described in patients with seawater exposures and immunocompromising conditions. We present a case of a previously healthy young woman who presented with fever and weakness 4 days after routine elective plastic surgery. Violaceous bullae were found from her bilateral breasts tracking down to her right thigh, corresponding to surgical sites. Despite linezolid and meropenem administration, the patient had hemodynamic instability, requiring intubation and vasopressor support. Necrotizing fasciitis was diagnosed and underwent emergent surgical debridement. Blood cultures grew *Shewanella algae*, with intraoperative cultures growing *S. algae*, *Pseudomonas aeruginosa*, and *Candida parapsilosis*. After 11 additional wound debridements and adjustment of antimicrobial therapy, her necrotizing fasciitis resolved; however, she was left with multiple deformities. Although the mechanism of acquisition of this infection is unclear, *S. algae* should be considered when treating necrotizing wound infections.

## INTRODUCTION

Since the discovery of *Shewanella* species in 1931, there have been more than 100 species identified.^1^ These Gram-negative, motile, variably biofilm-forming bacteria are generally found in warm and brackish environments, but they can be adaptive at surviving in less than ideal conditions.^2^^,^^3^ Brackish water is defined as a body of water that is a confluence of both fresh water and seawater. It is an area of harsh environments because of the changing salinity, and thus, organisms must adapt rapidly to these changes. Thus, surviving in brackish water is a challenge for these organisms. In humans, *Shewanella* favors infection of the bloodstream, skin, soft tissues, ears, nose, throat and peritoneal spaces. This is especially true of those who are immunocompromised and/or have been in the ocean or handled marine animals.^3^ Although several cases have been recorded in the United States, the majority had clear exposures to seawater and have been found primarily in Asia and Europe.^4^ As such, we present a case of an otherwise healthy 34-year-old woman with *Shewanella algae* necrotizing fasciitis and secondary bacteremia after breast implant excision, suction-assisted lipectomy, and bilateral gluteal fat grafting.^5^^,^^6^

## CASE REPORT

**Figure 1.**
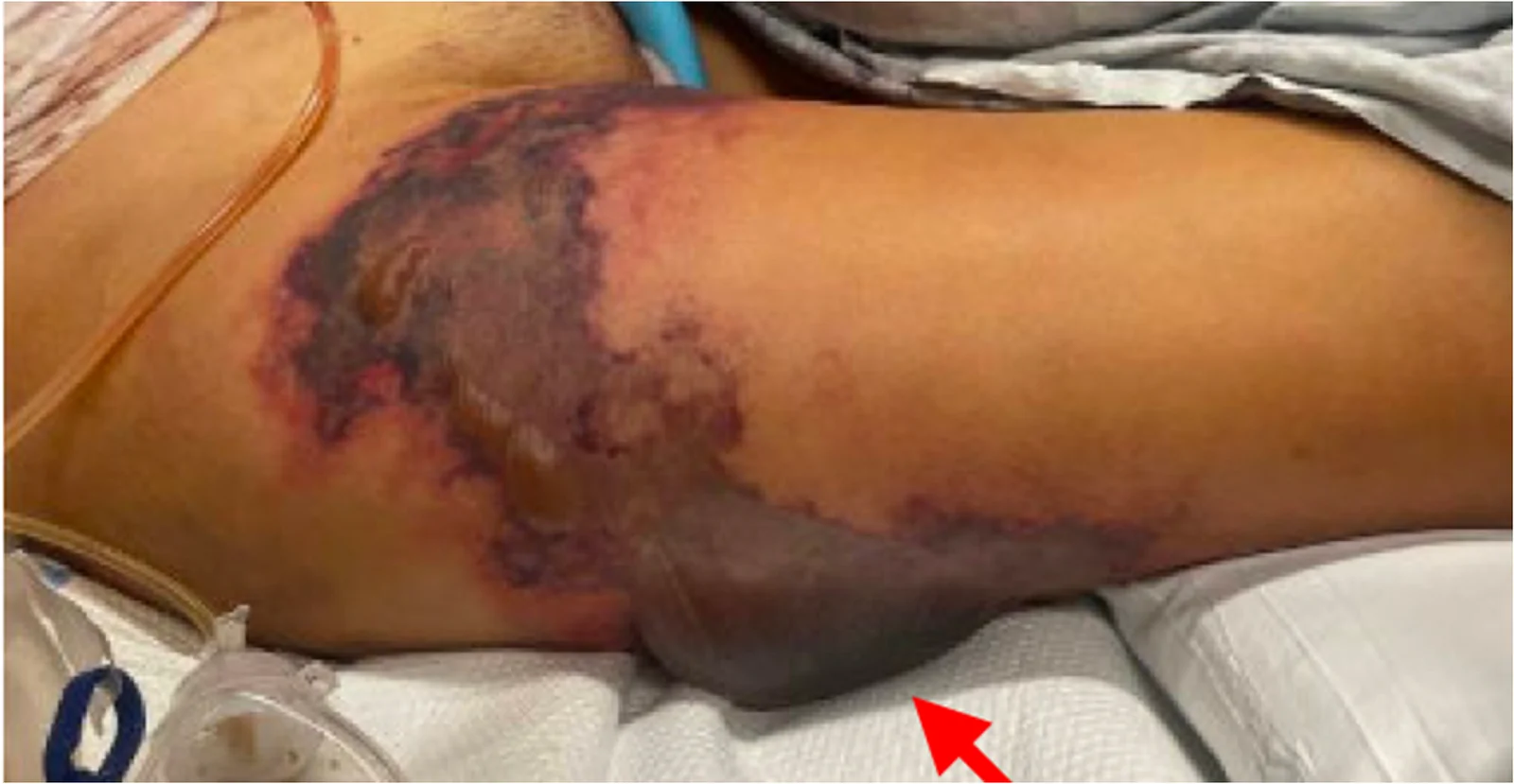
Right lateral thigh with a violaceous red rash with bullae throughout; the largest is indicated by the arrow.

**Figure 2.**
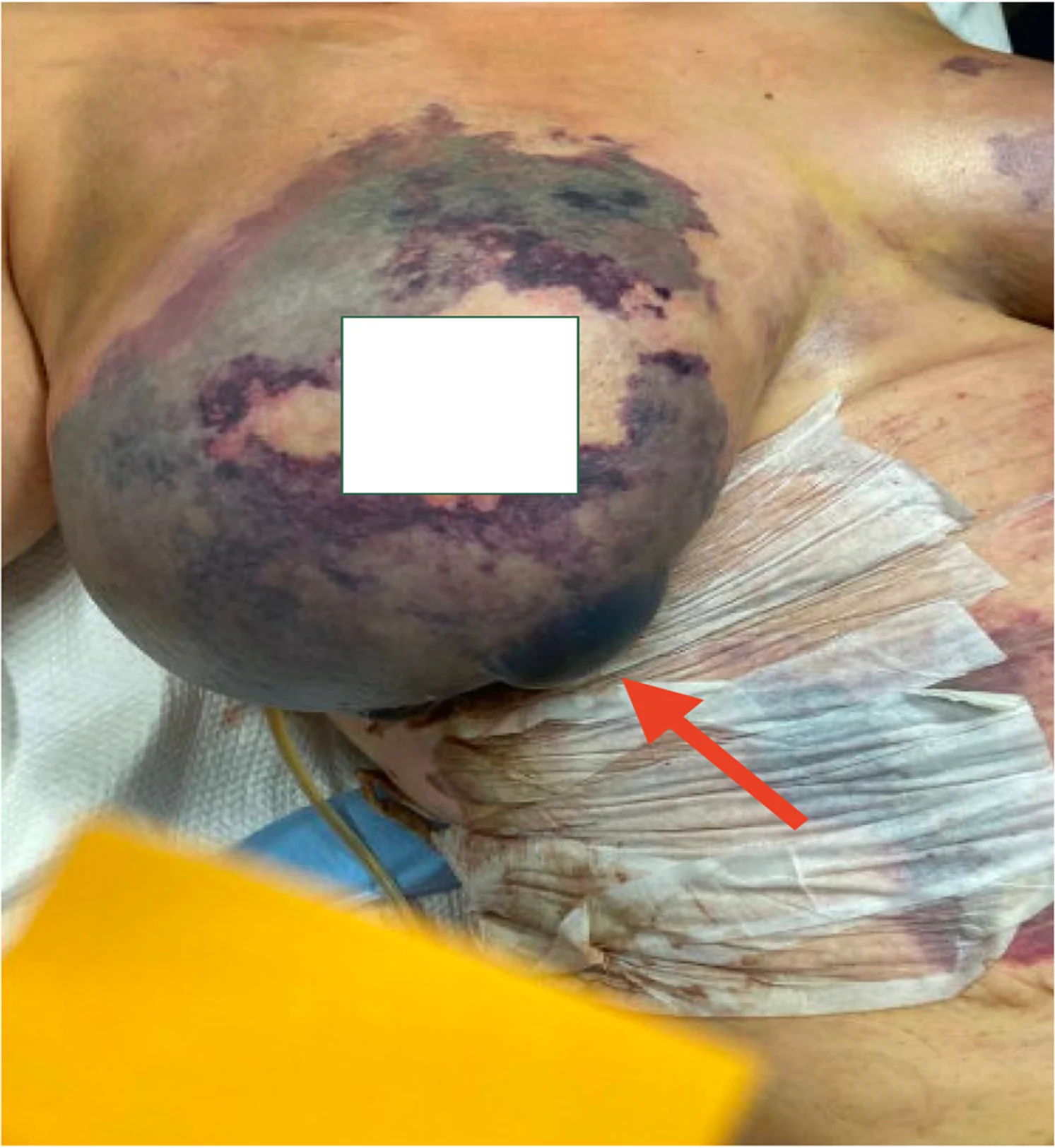
Right chest with the right breast encompassed by a violaceous rash and a bullae, which is indicated by the arrow.

A previously healthy 34-year-old woman presented to an outside hospital emergency room for generalized weakness, fever, and chills. Four days before presentation, she underwent elective breast implant excision and suction-assisted lipectomy of the abdomen with fat grafting to the breasts and buttocks by a plastic surgeon in southern Florida. The patient was transferred to our quaternary care facility because of the severity of her presentation. On examination, she was in severe pain that was unrelieved of analgesia; pertinent vitals included MAP of 40 to 50 mm Hg and febrile to 38.7°C. She had a diffuse, violaceous rash with tense bullae on her breasts bilaterally (Figure 1) that tracked down into the anterior right-side abdominal wall, right buttock, and lateral thigh (Figure 2). Laboratory data were significant for leukopenia of 2,520/*µ*L, thrombocytopenia of 47,000/*µ*L, hemoglobin of 7.9 g/dL, alanine aminotransferase of 142 U/L, aspartate aminotransferase of 215 U/L, creatine phosphokinase of 672 U/L, total bilirubin of 1.8 mg/dL, albumin of 1.2 g/dL, and high-sensitivity C-reactive protein of 45 mg/dL. She was admitted to the intensive care unit (ICU) given her unstable clinical status and vasopressor support requirement. Differential diagnosis included septic shock secondary to skin and soft-tissue infection versus toxic epidermal necrolysis. Board empiric antibiotic therapy was initiated with parenteral meropenem (1 g every 8 hours) and linezolid (600 mg every 12 hours).

General acute care and plastic surgery were consulted for possible intervention soon after arriving at the ICU. Because computed tomography imaging from the outside hospital was nondiagnostic for necrotizing infection, magnetic resonance imaging (MRI) of the chest, abdomen, pelvis, and thighs was recommended. En route to MRI, the patient became acutely altered and went into acute respiratory failure, requiring intubation. Magnetic resonance imaging showed no evidence of necrotizing fasciitis and no focal drainable abscess; however, there was evidence of intramuscular fluid along the musculature of the right femur, pelvis, and chest, with air tracking along the fascial planes in the chest. Surgical intervention was deferred; at that time, clinical presentation appeared to be consistent with postliposuction changes.

Over the next 12 hours, the patient continued to decline despite mechanical ventilation, broad-spectrum antibiotics, and vasopressor support. Her wounds began to drain malodorous fluid; the bullous rash spread further to her medial thigh, and she remained exquisitely tender. After surgical re-evaluation, the patient was taken emergently to the operating theater for irrigation, debridement, and deep wound cultures. Intraoperative notes described dusky, necrotic subcutaneous and superficial musculature with thin purulent material throughout.

On the second hospital day, four of four blood cultures from the outside hospital grew a Gram-negative bacillus identified as *S. algae* on matrix-assisted laser desorption/ionization time of flight (Table 1).^7^ In addition, her intraoperative wound cultures also grew Gram-negative rods that were later identified as *S. algae* (Table 1).^7^ Despite linezolid and meropenem parental therapy, the patient continued to have fevers, tachycardia, and increasing vasopressor requirements. A single dose of parenteral tobramycin (7 mg/kg; 510-mg dose) was given, and scheduled intravenous ciprofloxacin (750 mg every 12 hours) was added to the regimen given local *Shewanella* resistance patterns.

**Table 1:** Two isolates of *Shewanella*

Antibiotic name	*Shewanella algae*, Rare Growth and MIC (susceptibility)	*Shewanella algae* (second colony type), Light Growth and MIC (susceptibility)
Aztreonam	1 (susceptible)	1 (susceptible)
Cefepime	0.094 (susceptible)	0.25 (susceptible)
Ceftriaxone	0.75 (susceptible)	1.5 (susceptible)
Ciprofloxacin	0.25 (susceptible)	0.094 (susceptible)
Meropenem	0.38 (susceptible)	0.25 (susceptible)
Piperacillin/tazobactam	0.75 (susceptible)	1.5 (susceptible)
Trimethoprim/ sulfamethoxazole	0.19 (susceptible)	0.064 (susceptible)

MIC = minimum inhibitory concentration. The information in column 2 is from blood cultures at an outside hospital. The information in column 3 is from deep intraoperative leg wound cultures at our institution. The bacterium was identified by matrix-assisted laser desorption/ionization time of flight with MIC levels, and therefore, sensitivities were determined by the Clinical and Laboratory Standards Institute non-Enterobacterales MIC cutoffs.^7^

Over the next 48 hours, she returned to the operating theater twice more for further exploration and debridement. On hospital day 4, the patient was able to be weaned off vasopressors and extubated successfully. At that time, intraoperative cultures grew *S. algae* and *Pseudomonas aeruginosa* (susceptibilities are shown in Table 1). Antimicrobials were adjusted to monotherapy with parenteral cefepime (2 g every 8 hours). An additional intraoperative culture grew *Candida parapsilosis*, for which she was placed on micafungin intravenously (100 mg daily).

After 44 days since admission, 12 debridements, and multiple autologous skin grafts, the patient was discharged to a rehabilitation center. She was administered an additional 14 days of parenteral cefepime and oral fluconazole. The patient made a full recovery but has been left with multiple cutaneous deformities that are still being managed by plastic surgery.

## DISCUSSION

Cases of *Shewanella* human infection have the highest report rates in southern Europe, Southeast Asia, and southern Africa during the hot summer months.^8^ Rising sea temperatures and increases in global travel may contribute to a growing number of cases in the United States. Marine scientists have already demonstrated that the ocean has not only become warmer but also, more acidic and deoxygenated. This selective pressure has changed microbial populations and may increase the number of facultative anaerobes, such as *Shewanella*.^9^

In our patient’s case, there are no clear risk factors for this type of infection. She denied any marine/sea exposures or recent travel out of the United States. In addition, she had no obvious immunodeficiencies; her original procedure was completed at a licensed ambulatory surgical center, and she completed her prescribed postoperative antibiotics. Some feasible routes of infection could include sterile saline contamination, biofilm formation on surgical equipment, ingestion of seafood with translocation, or exposure of the postoperative sites to tap water.

*Shewanella* can create biofilms, use adhesions, and produce hemolysin to evade the host’s immune system. In addition, *Shewanella* has been described as the potential reservoir for certain antibiotic-resistance genes.^10^ In particular, *Shewanella* has the genetic code for oxacillinase class D-lactamase within it. When expressed, this enzyme degrades beta-lactam antibiotics and therefore, makes the infection more difficult to treat.^11^ Many of its characteristics resemble those of *Vibrio* species, including overall clinical manifestations in humans, underscoring its potential public health significance and the importance of continued attention and surveillance. However, unlike *Vibrio* necrotizing fasciitis, our patient had a polymicrobial infection with *Pseudomonas* and *Candida*. Although *Candida* could have been a contaminant from the surrounding skin, it is unclear whether the *Pseudomonas* was an opportunistic pathogen obtained from hospitalizations or was obtained similarly with *Shewanella*.

Currently, this remains the only reported case of *Shewanella*-induced infection associated with plastic surgery. Although *Staphylococcus* species remain the leading bacteria associated with plastic surgery site infections, clinicians should consider *Shewanella* when treating aggressive skin and soft-tissue infections, regardless of risk factors.
